# Left ventricular haemodynamic effects in a patient with endocarditis

**DOI:** 10.1007/s12471-021-01616-0

**Published:** 2021-08-17

**Authors:** M. A. W. Habets, S. Bouwmeester

**Affiliations:** Department of Cardiology, Catharina Hospital Eindhoven, Eindhoven, The Netherlands

## Answer

Echocardiography showed severe aortic regurgitation (AR) with diastolic mitral regurgitation (MR). Under normal conditions, a negative pressure gradient from left ventricle (LV) to left atrium (LA) during diastole keeps a forward blood flow through the mitral valve. Diastolic MR occurs due to reversal of the pressure gradient between LV and LA. Severe acute AR causes volume overload and high left ventricular end-diastolic pressure (LVEDP). When the LVEDP rises above the atrial pressure, the mitral valve leaflets approach each other. This results in incomplete mitral valve closure and end-diastolic MR before the LV contracts [1]. The diastolic regurgitant volume is usually small due to a low ventricle-atrial pressure gradient, with a far lower flow velocity than systolic MR [1, 2].

**Fig. 1:**
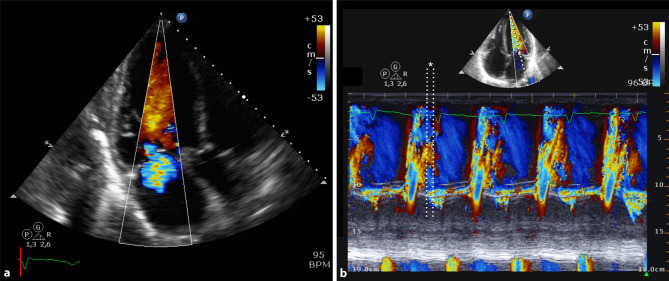
**a** Colour flow Doppler still frame from apical three-chamber view with diastolic MR; **b** Colour M‑mode of transmitral flow from the apical four-chamber view with diastolic MR (* indicates the Q‑Mc time). *MR* mitral regurgitation

Fig. 1 shows the diastolic MR. The first white line in panel B indicates the start of the diastolic MR which merges into a systolic MR at the second white line. The time between those lines is the Q‑Mc time, the time between the Q wave and closure of the mitral valve. Because of the high LVEDP in this patient, the mitral valve closes during diastole before LV contraction, and the Q‑Mc time becomes negative (−80 ms).

It is important to recognise diastolic MR in patients with aortic regurgitation since it implies a severe haemodynamic state that requires emergency surgery. The patient underwent a successful emergency aortic valve replacement. The culture of the native aortic valve was positive for *Capnocytophaga canimorsus*, a commensal bacterium in the saliva of dogs. Antibiotics were continued intravenously for 6 weeks after surgery. The patient recovered after a rehabilitation period and the diastolic MR disappeared.
